# Work in the New Field

**Published:** 1915-03

**Authors:** T. D. Crothers


### EDITORIAL DEPARTMENT.

MRS. F. E. DANIEL, Publisher and Managing Editor.
DR. R. H. L. BIBB, Associate Editor.

CONTRIBUTING EDITORS:

Dr. H. Sheridan Baketel, New York, editor *Medical Times;* Dr. M. H. Boerner, Austin, Texas; Dr. G. Henri Bogart, Paris, Ill.; Dr. M. M. Carrick, Dallas, Texas; Dr. Edward H. Carey, Dean Medical Department, Baylor University, Dallas, Texas; Dr. W. L. Crosthwaite, Waco, Texas; Dr. T. D. Crothers, Hartford, Conn.; Dr. H. W. Cummings, Hearne, Texas; Dr. Oscar Dowling, New Orleans, President Louisiana State Board of Health; Havelock Ellis, M. D., London, Eng.; Dr. May Agnes Hopkins, Dallas, Texas; Dr. A. W. Fly, Galveston, Texas; Dr. G. B. Foscue, Waco, Texas; Dr. E. S. Goodhue, Holualoa, Kona, Hawaii; Dr. M. B. Grace, Seguin, Texas; Dr. Joe Gilbert. Austin, Texas; Dr. Charles H. Hughes, St. Louis, editor *Alienist and Neurologist;* Dr. James G. Kiernan, Chicago; Dr. George H. Lee, Galveston, Texas; Dr. John T. Moore, Houston, Texas; Dr. Frank Paschal, San Antonio, Texas; Dr. John Preston, Austin, Texas, Superintendent State Lunatic Asylum; Dr. G. Wilse Robinson, Kansas City, Mo.; Dr. Bacon Saunders, Fort Worth, Texas; Dr. Allen J. Smith, Philadelphia, Medical Department, University of Pennsylvania; Dr. Z. T. Scott, Austin, Texas; Dr. Ralph Steiner, Austin, Texas, President Texas State Board of Health; Dr. Walter S. Sutton, Kansas City, Mo.; Dr. John S. Turner, Dallas, Texas; Dr Charles Venable, San Antonio, Texas.

---

### Work in the New Field.

BY T. D. CROTHERS.

An anxious mother appealed to a physician to know what she should do in the training of her two boys. The father had killed himself by drink a few years before, and the mother realized that these little boys were not safe unless trained along special lines. The advice was to keep them in the open air as much as possible, avoid every possible stimulant of coffee, teas, meat, and condiments and quietly teach them that alcohol was a poison. For several years the mother constantly appealed to the physician for advice, and this attracted his attention to the laws of heredity and the possibility of predisposition and how the first beginnings of abnormal appetites could be restrained.

Both boys grew strong and vigorous and grew up thoroughly temperate in every respect. In contrast with this was the appeal of a man of wealth to his family physician to know how he could bring up his son and daughter, to avoid the mistakes of their mother, who, after drinking many years, became a drug taker and died.

The advice was not to say much about the alcoholic question. If he condemned alcohol bitterly it would only attract and stimulate the children's minds to try it. If they showed any weakness of any kind to call him, and he would give them appropriate medicines which were really tinctures made palatable.

The father, who had been a drinking man, and realized the danger of heredity, left the matter entirely to the physician and housekeeper. By and by the eldest, a boy, became intoxicated. The girl was obliged to take drugs every month and displayed a great many abnormalities of appetite.

The father still trusted to the physician, who still blundered. The result was as the father feared. The boy was expelled from college for drinking, and the girl became an invalid. Had the last physician realized the positiveness of the laws of heredity and laid down some rule of training and hygienic preventive measures, both children would have been saved.

This new field of practice he did not know. He might have been a good surgeon, clever in diagnosis, but he was densely ignorant of the progress made concerning alcohol and its effects on the system. Heredity, which he should study, was unknown to him and he lived believing that the teachings of the last century were final truths, and no doubt he will die, largely the result of his own misconceptions.

The new field is dawning and every physician ought to realize the power of heredity and the force of energies in childhood and infancy that will permanently mar and break up all future health and vigor.

The value of proper advice to parents and teachers from infancy upward is far more potent and practical than what to do when disease appears.

In one case it is forecasting and applying preventive measures. In the other it is palliative and trying to lessen the intensity of the disease processes.

Start the body and mind right, direct it along healthy channels and in accord with the great laws of nature, and the aftergrowth will be complete. Neglect the mind and surroundings and the conditions that are abnormal, and the future of disaster can be predicted with absolute certainty.

Mothers intuitively understand this in a measure, but physicians should make it a part of their daily practice to give counsel and direction in the earliest stages of childhood. If parents were trained to depend on the physician for means and measures that would retard disease tendencies and develop strong, vigorous growth, they would have an enormous practice. The physicians do not prepare themselves for this field, hence are not consulted.

These are the lines along which the highest and best medical skill can be exercised.

---

Dr. J. M. Gore has left Blum and moved to Lubbock.

